# Molecular Epidemiology and *In-Vitro* Antifungal Susceptibility of *Aspergillus terreus* Species Complex Isolates in Delhi, India: Evidence of Genetic Diversity by Amplified Fragment Length Polymorphism and Microsatellite Typing

**DOI:** 10.1371/journal.pone.0118997

**Published:** 2015-03-17

**Authors:** Shallu Kathuria, Cheshta Sharma, Pradeep Kumar Singh, Puneet Agarwal, Kshitij Agarwal, Ferry Hagen, Jacques F. Meis, Anuradha Chowdhary

**Affiliations:** 1 Department of Medical Mycology, Vallabhbhai Patel Chest Institute, University of Delhi, Delhi, India; 2 Department of Pulmonary Medicine, Vallabhbhai Patel Chest Institute, University of Delhi, Delhi, India; 3 Department of Pulmonary Medicine, Rajan Babu Institute of Pulmonary Medicine and Tuberculosis, Delhi, India; 4 Department of Medical Microbiology and Infectious Diseases, Canisius-Wilhelmina Hospital, Nijmegen, The Netherlands; 5 Department of Medical Microbiology, Radboudumc, Nijmegen, The Netherlands; Leibniz Institute for Natural Products Research and Infection Biology- Hans Knoell Institute, GERMANY

## Abstract

*Aspergillus terreus* is emerging as an etiologic agent of invasive aspergillosis in immunocompromised individuals in several medical centers in the world. Infections due to *A*. *terreus* are of concern due to its resistance to amphotericin B, *in vivo* and *in vitro*, resulting in poor response to antifungal therapy and high mortality. Herein we examined a large collection of molecularly characterized, geographically diverse *A*. *terreus* isolates (n = 140) from clinical and environmental sources in India for the occurrence of cryptic *A*. *terreus* species. The population structure of the Indian *A*. *terreus* isolates and their association with those outside India was determined using microsatellite based typing (STR) technique and Amplified Fragment Length Polymorphism analysis (AFLP). Additionally, *in vitro* antifungal susceptibility of *A*. *terreus* isolates was determined against 7 antifungals. Sequence analyses of the calmodulin locus identified the recently described cryptic species *A*. *hortai*, comprising 1.4% of *Aspergillus* section *Terrei* isolates cultured from cases of aspergilloma and probable invasive aspergillosis not reported previously. All the nine markers used for STR typing of *A*. *terreus* species complex proved to be highly polymorphic. The presence of high genetic diversity revealing 75 distinct genotypes among 101 Indian *A*. *terreus* isolates was similar to the marked heterogeneity noticed in the 47 global *A*. *terreus* population exhibiting 38 unique genotypes mainly among isolates from North America and Europe. Also, AFLP analysis showed distinct banding patterns for genotypically diverse *A*. *terreus* isolates. Furthermore, no correlation between a particular genotype and amphotericin B susceptibility was observed. Overall, 8% of the *A*. *terreus* isolates exhibited low MICs of amphotericin B. All the echinocandins and azoles (voriconazole, posaconazole and isavuconazole) demonstrated high potency against all the isolates. The study emphasizes the need of molecular characterization of *A*. *terreus* species complex isolates to better understand the ecology, acquisition and transmission of this species.

## Introduction

Invasive aspergillosis (IA) is a devastating and difficult to manage disease, which is associated with significantly high morbidity and mortality, especially in immunocompromised patients with haematological malignancy or recipients of allogeneic hematopoietic stem cell transplantation [1– 3]. Furthermore, other forms of aspergillosis such as aspergilloma, chronic pulmonary aspergillosis (CPA) and allergic bronchopulmonary aspergillosis (ABPA) can cause considerable morbidity and mortality in immunocompetent or mildly immunocompromised hosts [4]. While *Aspergillus fumigatus* is the most common causative agent of IA, *Aspergillus terreus* remains the third most important etiologic agent of IA [5–7]. Interestingly, *A*. *terreus* appears to be the commonest cause of infection in some medical centers, particularly in Houston, Texas and Innsbruck, Austria [8, 9]. Infections due to *A*. *terreus* are worrisome due to its *in vivo* and *in vitro* resistance to amphotericin B (AMB) and are thus associated with a lower rate of response to antifungal therapy and a higher rate of IA-associated mortality (51% versus 30%) compared with non-*terreus* species of *Aspergillus* [8–13].

Recently, Samson et al. (2011) [14], using a polyphasic approach, described seven lineages among *A*. *terreus* isolates. They proposed 7 species in *Aspergillus* section *Terrei* namely *A*. *terreus sensu stricto*, *A*. *alabamensis*, *A*. *floccosus*, *A*. *neoafricanus*, *A*. *aureoterreus*, *A*. *hortai* and *A*. *pseudoterreus* [14]. Although the new species are defined in the *A*. *terreus* species complex, molecular studies exploring the population structure of this important fungal pathogen are limited *vis-à-vis* that of *A*. *fumigatus* [15–17]. Previously only two studies, originating from the USA had explored the population structure of global *A*. *terreus* isolates [18, 19]. Balajee et al. [18] using multi-locus comparative sequence analysis of three genes reported the existence of a single, globally distributed *A*. *terreus* population in a collection of 94 clinical and environmental isolates. However, Neal et al. [19] in 2011 genotyped 117 *A*. *terreus* isolates from the USA and Europe by using Inter-Simple Sequence Repeat (ISSR) PCR and demonstrated that one clade comprised exclusively of isolates from Europe and another was enriched with isolates from the USA. Overall, the data on genetic variability within *A*. *terreus* is inadequate due to lack of application of reliable methods. We aimed to study the population structure of a large collection of 140 molecularly characterized *A*. *terreus* isolates from various hospitals and the environment in Delhi, India and its adjoining regions, using a robust microsatellite based typing technique (referred as short tandem repeat; STR typing) and Amplified Fragment Length Polymorphism (AFLP) analysis. Furthermore, the association between isolates from India and those outside of India was studied. Additionally, *in vitro* antifungal susceptibility of *A*. *terreus* isolates against medical triazoles, echinocandins and AMB using CLSI M38-A2, was determined to examine if any specific antifungal susceptibility pattern correlated with a particular genetic lineage. The data obtained by genotyping methods revealed the presence of a recently delineated cryptic species, *A*. *hortai* in the *Aspergillus* section *Terrei*, as an etiologic agent of aspergilloma and IA, not reported previously.

## Materials and Methods

### Ethics Statement

All necessary permits were obtained for the described field studies. The study was approved by the V.P. Chest Institute’s (VPCI) Ethics Committee, and a written informed consent was taken from all subjects. The ethical clearance for field studies of Central park, VPCI was obtained by V.P. Chest Institute’s Ethics Committee. The permission for collection of soil from privately owned agricultural fields were obtained from the owner (JK, Haryana).

### Fungal isolates and their morphological characterization

A total of 140 *A*. *terreus* isolates including 128 clinical isolates collected from 6 hospitals in Delhi and 12 environmental isolates, during 2009–2014 were analyzed. The details of Indian clinical and environmental isolates are included in S1 Table and global isolates used in this study are included in S2 Table. Briefly, clinical isolates originated from patients with chronic respiratory disorders, IA, ABPA, allergic fungal rhinosinusitis (AFRS), and CPA. The 12 *A*. *terreus* soil isolates were selected from a collection of environmental isolates, processed and stocked during an ongoing survey of azole resistant *A*. *fumigatus* [20]. Of these, two *A*. *terreus* isolates originated from the central park surrounding V. P. Chest Institute, and the remaining 10 isolates were from a rose garden (n = 3) and agricultural fields of rice (*Oryza sativa*, n = 3), red chilli (*Capsicum annuum*, n = 2), fenugreek (*Trigonella foenum-graecum*, n = 1) and wheat (*Triticum aestivum*, n = 1) in Delhi and Haryana, India.

All of the isolates were stored at -70°C in glycerol. Preliminary species identification was based on colony colour and morphology of the isolates on Czapek dox agar plates incubated at 28°C for 7 days.

### Molecular identification by (*Cmd*) gene sequencing and Phylogenetic analysis

All phenotypically characterized *A*. *terreus* isolates were confirmed by sequencing of the *Cmd* gene. DNA extraction was done as described previously [21]. Briefly, the DNA was extracted by subjecting *A*. *terreus* spores to bead beating in the presence of extraction buffer (0.2 M Tris-HCl 10 mM EDTA, 0.5 M NaCl, 1% SDS) followed by the phenol, chloroform and isoamyl alcohol (25:24:1) extraction and ethanol precipitation. The extracted DNA was subjected to amplification of part of the *Cmd* gene with primers cmd5 (5’-CCGAGTACAAGGAGGCCTTC-3’) and cmd6 (5’-CCGATAGAGGTCATAACGTGG-3’) [22]. Additionally, a set of distinct 12 *A*. *terreus* isolates revealing a separate clade in *Cmd* phylogenetic analysis were also characterized using *β-tubulin* gene primers Bt2a (5’-GGTAACCAAATCGGTGCTGCTTTC-3’) and Bt2b (5’-ACCCTCAGTGTAGTGACCCTTGGC-3’) [23].

The amplification was followed by purification of the amplified product using Wizard SV Gel and PCR Clean-up System (Promega, Fitchburg, WI, USA) and sequencing on an ABI 3130XL genetic analyzer (Applied Biosystems, Foster City, CA) using the Big Dye terminator kit (v3.1, RR-100; Applied Biosystems) [21]. DNA sequences were analyzed with Sequencing Analysis software version 5.3.1 (Applied Biosystems). *Cmd* and *β-tubulin* gene sequences were subjected to BLAST searches at GenBank (http://www.ncbi.nlm.nih.gov/BLAST/Blast.cgi) and CBS database (http://www.cbs.knaw.nl/Collections/BioloMICSSequences.aspx?file=all). Sequence-based species identification was defined by ≥99% similarity. For phylogenetic analyses, the *Cmd* and *β- tubulin* gene sequences of the type and reference *A*. *terreus* isolates were retrieved from NCBI database. All the sequences were aligned with ClustalW program (version 1.82), and the final alignments were edited manually. A maximum likelihood tree based on *Cmd* and *β-tubulin* gene sequences using 2000 bootstrap replications were constructed using MEGA version 5 [24].

### Genotyping of *A*. *terreus* isolates

A set of 122 *A*. *terreus* isolates (110 clinical, 12 environmental) based on *Cmd* sequence analysis were selected for genotypic analysis using AFLP and STR technique. Of 140 isolates, 18 exhibiting >99% sequence similarity and mainly colonizers were excluded for AFLP and STR typing.

### Amplified fragment length polymorphism analysis

About 50 ng of genomic DNA was subjected to restriction ligation procedure using *HpyCH4IV* and *MseI* restriction enzymes (New England Biolabs, Beverly, MA, USA) and complementary adaptors as described previously [25]. The restriction-ligation reaction was diluted by adding 80 μl Tris/HCl (pH 8.3) buffer. One microliter of this diluted product was amplified in a final volume of 25 μl, using selective primers HpyCH4IV-C (5′-FLU-GTAGACTGCGTACCCGTC-3′) and MseI-TGAG (5′-GATGAGTCCTGACTAATGAT-3′). One microliter of the 10× diluted amplicon was added to a mixture of 8.9 μl water and 0.1 μl LIZ600 internal size marker (Applied Biosystems), followed by heating the diluted sample for 1 min to 95°C and subsequent fragment analysis on an ABI 3500xL Genetic Analyzer (Applied Biosystems). A raw data analyses was done using Bionumerics v6.0 (Applied Maths, Sint-Martens-Latem, Belgium) and a dendrogram using standard Pearson and unweighted pair group method with arithmetic mean (UPGMA) was generated. The reference/type strains of *Aspergillus terreus* CBS 601.65^T^ (soil, Connecticut); *A*. *terreus* var. *floccosus* CBS 116.37^T^ (syn. *A*. *floccosus*, waste cloth, China) and *A*. *terreus* var. *africanus* CBS 130.55^T^ (syn. *A*. *neoafricanus*, soil, Ghana) were included for AFLP analysis.

### Microsatellite analysis

A total of 122 *A*. *terreus* isolates were subjected to microsatellite typing using a panel of nine short tandem repeats (STR) to evaluate the genetic relatedness between the isolates. Twenty-one isolates revealing no amplification at >2 loci after repeated attempts were excluded from the analysis. Thus, STR analysis was carried out for 101 *A*. *terreus* isolates (91 clinical, 10 environmental). Three di-, tri- or tetranucleotide repeat markers, described previously, were amplified using three multiplex PCRs, namely, M2, M3 and M4, respectively [26]. One of the primers for each marker was labeled at the 5’end with either carboxyfluorescein (FAM), dimethoxyfluorescein (JOE) or tetramethylrhodamine (TAMRA). Optimization of primer concentration and PCR conditions were carried out as described previously [26]. Briefly, the reaction was run with an initial denaturation step of 95°C for 10 min followed by 30 cycles of 30 s denaturation, 30 s of annealing at 60°C and 1 min extension at 72°C and a subsequent final elongation at 72°C for 10 min.

The PCR products, so obtained, were diluted 100-fold with distilled water and 1 μl of this diluted product was combined with 0.1 μl of CC-500-ROX marker (Promega). The amplicons were separated by size and detected on an ABI3500xL Genetic Analyzer platform equipped with a 24-capillary array (Applied Biosystems) as per manufacturer recommendations. Repeat numbers in each marker was assigned by using *A*. *terreus* NIH 2624 as reference. Additionally, isolates from USA (n = 20); Europe including France (n = 4), Germany (n = 2), Italy (n = 2), Norway (n = 2), Slovenia (n = 1), Spain (n = 4), the Netherlands (n = 5); and China (n = 1), New Zealand (n = 1), Panama (n = 1), Papua New Guinea (n = 2), Taiwan (n = 1) and Thailand (n = 1) were used for the analysis. A minimum spanning tree was generated to illustrate the genotypic diversity among clinical *Aspergillus terreus* isolates from India and those from outside India.

### Antifungal Susceptibility Testing (AFST)

The *in vitro* susceptibility testing of 140 *A*. *terreus* isolates to all the antifungals was done using microbroth dilution CLSI M38-A2 document [27]. The drugs tested included itraconazole (ITC, Lee Pharma, Hyderabad, India, and Janssen Research Foundation, Beerse, Belgium), voriconazole (VRC, Pfizer Central Research, Sandwich, Kent, U.K.), isavuconazole (ISA, Basilea Pharmaceutica International AG, Basel, Switzerland), posaconazole (POS, Merck, Whitehouse Station, NJ, USA), amphotericin B (AMB, Sigma-Aldrich, Germany), caspofungin (CFG, Merck), micafungin (MFG, Astellas Toyama Co. Ltd., Japan) and anidulafungin (AFG, Pfizer). Drug-free and mold-free controls were included and microtitre plates were incubated at 35°C and MIC readings were taken after 48 hr for azoles and AMB and 24 hr for echinocandins. CLSI recommended quality control strains, *Candida krusei*, ATCC6258 and *Candida parapsilosis*, ATCC22019 and reference strains *Aspergillus fumigatus*, ATCC204305 and *Aspergillus flavus*, ATCC204304 were included. MIC end points for all the drugs except echinocandins were defined as the lowest concentration that produced complete inhibition of growth *vis-à-vis* the hyphal growth in the control well. Minimum effective concentration (MECs) of echinocandins were defined as the lowest drug concentrations that allowed the growth of small, rounded, degenerated hyphae *vis-à-vis* the growth in the control well. The AFST results for *A*. *terreus* in this study were analyzed using recently described epidemiological cutoff values (ECVs) ITC, 1 mg/L; VRC, 1mg/L; POS, 0.5 mg/L; ISA, 1 mg/L; AMB, 8 mg/L and CFG, 0.25 mg/L [28–30].

### Patient details

The records of patients with a positive culture for *A*. *terreus* were reviewed. The data collected included demographics, information on underlying disease/risk factors, the clinical disease entity attributable to *A*. *terreus* such as IA, aspergilloma, ABPA, AFRS, CPA or colonization of the respiratory tract. IA was defined as probable or definite according to the criteria of the European Organization for Research and Treatment of Cancer Mycoses Study Group (EORTC/MSG) [31]. CPA was diagnosed by chronic duration of clinical symptoms (>3 months), progressive pulmonary lesions with or without cavitation, precipitating antibodies to *A*. *terreus* in serum, mycological evidence of fungal presence, with or without the background of immunocompromising factors (diabetes mellitus, leukemia, chronic steroid therapy etc). Aspergilloma were noted to be present as a mobile, intra cavitary mass with an air crescent sign in the periphery with a positive culture of respiratory specimens for *A*. *terreus* and serum precipitins against the fungus. ABPA was diagnosed by a combination of clinical, mycoserologic and radiological features as proposed by Rosenberg and Patterson [32]. AFRS was diagnosed by the deShazo and Swain criteria, which included type 1 hypersensitivity, nasal polyposis, characteristic computed tomography findings, eosinophillic mucin without invasion and a positive fungal stain of sinus contents [33].

### Ouchterlony’s immunodiffusion test

The patients sera were tested for precipitins against in-house prepared culture filtrate antigen of *A*. *terreus* by Ouchterlony’s immunodiffusion test as described previously [34].

### Specific IgE estimation by Enzyme-linked Immunosorbent assay (ELISA)

*Aspergillus terreus* extract was in-house prepared and specific IgE in patients sera was determined by ELISA as described previously [34]. Briefly, a microtitre plate (Nunc-ImmunoTM modules, Roskilde, Denmark) was coated with *A*. *terreus* extract (1 μg/100 μl/well) in carbonate buffer, pH 9.6, blocked with 3% defatted milk, washed with PBST (0.1 M PBS containing 0.2% Tween 20) and incubated with patient’s serum or control sera (1: 10 v/v) at 4°C overnight. The plate was then incubated with 1: 1000 v/v anti-human-IgE peroxidase (Sigma, USA), colour developed with o-phenylenediamine and read at 492 nm in an ELISA reader. ELISA was performed in triplicate and the mean of three readings was considered for analysis. Normal human sera (pooled) were used as negative control.

## Results

All *A*. *terreus* isolates (n = 140) originating from clinical and environmental sources showed yellowish-brown to cinnamon-brown colonies consisting of a dense felt of conidiophores on Czapek dox agar plates. Dense columnar conidial heads with smooth-walled hyaline conidiophore stipes along with biseriate conidiogenous cells were observed in the lactophenol cotton blue mounts.

### Molecular Identification and Phylogenetic analysis

Of the 140 *A*. *terreus* isolates sequenced for *Cmd* gene, 138 (GenBank accession nos. KM386696- KM386816 and KM458096-KM458112) showed 99% homology (query coverage ranging from 98–100%) with *A*. *terreus* isolates in GenBank (accession nos. KJ146014, JF927632, EU147582) whereas the remaining two isolates viz., VPCI 317/P/11 and VPCI 906/12 (GenBank accession nos. KM386817 and KM386818) showed 99% homology with *A*. *hortai* isolates from Greece and Czech Republic, (accession nos. JQ806413 and FR837976).

**Fig 1:**
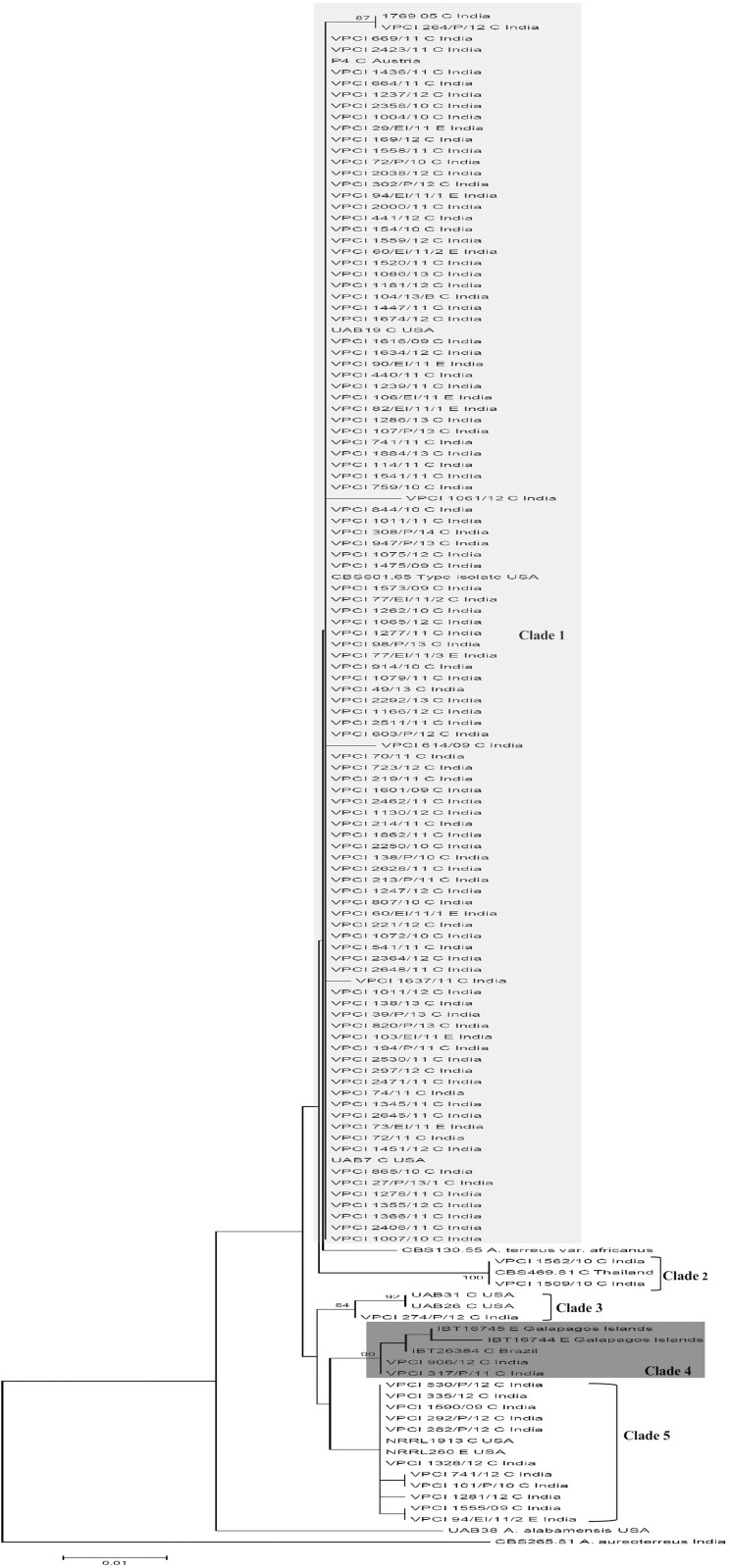
Phylogenetic tree based on partial sequence of *calmodulin* gene using maximum likelihood analysis depicting intraspecies variation among *A*. *terreus* isolates. *Aspergillus terreus* (CBS 601.65^T^), *Aspergillus alabamensis* (UAB38), *A*. *terreus* var. *africanus* (syn. *A*. *neoafricanus* CBS 130.55^T^), *A*. *aureoterreus* (CBS 265.81^T^), *A*. *hortai* (IBT16744, IBT16745 and IBT26384) of *A*. *terreus* section *Terrei* were taken as outliers for the analysis. Bootstrap values are shown above the branches. Environmental isolates are denoted by E, and clinical isolates are denoted by C.

The Maximum likelihood tree of all the Indian *A*. *terreus* isolates was generated from 491 contiguous bases of aligned sequences of *Cmd* gene region. For phylogenetic analysis, *Cmd* gene sequence of *A*. *terreus* reference/ type strains (n = 14), *A*. *alabamanesis* (n = 1), *A*. *terreus* var. *africanus* (syn. *A*. *neoafricanus*) (n = 1) and *A*. *aureoterreus* (n = 1) of section *Terrei* were retrieved from GenBank (Fig. 1). The *Cmd* phylogenetic tree (Fig. 1) yielded 5 distinct clades and enabled the differentiation of newly described *A*. *hortai* species of *A*. *terreus* species complex. Primarily, clade 1 represented 75% of Indian *A*. *terreus* isolates, which clustered with the type strain, *A*. *terreus* CBS 601.65^T^and *A*. *terreus* var. *africanus* (syn. *A*. *neoafricanus* CBS 130.55^T^). Also, barring a solitary environmental isolate, all other 11 environmental isolates clustered with the clinical isolates in clade 1. Indian clinical and environmental isolates exhibited 97–100% sequence similarity among themselves. Furthermore, previously reported Indian *A*. *terreus* clinical isolate (1769–05) by Balajee et al., [18] also fell in clade 1 and revealed 100% similarity with the present study clinical isolate VPCI 264/P/12. A smaller clade 2, comprising two clinical isolates (VPCI 1509/10 and VPCI 1562/10) and a solitary *A*. *terreus* isolate (CBS469.81) from a cardiac valve of a patient from Thailand [18] showed 100% similarity with each other. Similarly, clade 3 represented only 3 isolates comprising 2 clinical isolates (UAB31 and UAB26) from USA originating from BAL and sputum [18] and one clinical Indian isolate (VPCI 274/P/12) from a BAL of an ABPA patient. *Aspergillus hortai* represented a distinct clade 4 comprising two Indian clinical isolates (VPCI 906/12 and VPCI 317/P/11) and 2 environmental *A*. *hortai* isolates (IBT16744 and IBT16755) from the Galapagos Islands and one clinical isolate (IBT26384) from Brazil [18]. The two Indian *A*. *hortai* isolates revealed 100% sequence similarity with each other and exhibited 99% sequence similarity with Brazilian and Galapagos islands isolates. Noticeably, clade 5 was distinct from rest of *A*. *terreus* isolates and included 11 Indian isolates (10 clinical, 1 environmental) and 2 isolates (clinical and environmental) from the USA. Also, the clade 5 isolates *β tubulin* gene region sequences analyzed by maximum likelihood revealed the same clustering pattern and well-defined group with good bootstrap value (99%).

### Amplified Fragment Length Polymorphism

**Fig 2:**
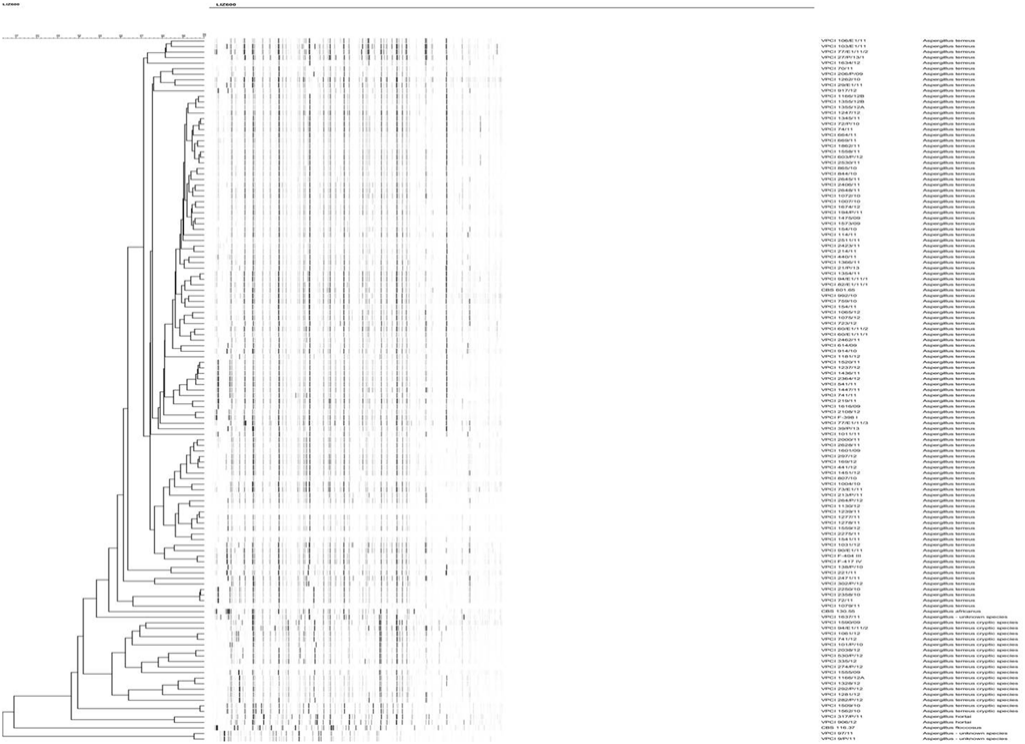
Amplified fragment length polymorphism analysis showing genotypic diversity among 122 clinical and environmental Indian A. terreus isolates. *Aspergillus terreus* (CBS 601.65^T^), *A*. *terreus* var. *africanus* (syn. *A*. *neoafricanus* CBS 130.55^T^), *A*. *terreus* var. *floccosus* (syn. *A*. *floccosus* CBS 116.37^T^) were used for the analysis. The dendrogram was constructed by using UPGMA (unweighted pair group method with averages) in combination with the Pearson correlation coefficient and was restricted to fragments of 60–400 bp. Scale bar indicates the percentage similarity.

The AFLP fingerprint analysis showing genotypic diversity among 122 (clinical, 110; environmental, n = 12) Indian *A*. *terreus* isolates is depicted in Fig. 2. The isolates could be distinguished based on the distinct banding patterns. UPGMA analysis (Fig. 2) showed that the isolates grouped into four clusters. Majority of isolates (n = 103) that formed clade 1 in *Cmd* phylogenetic analysis showed >95% similarity between the fingerprints including type strains (CBS 601.65^T^, CBS 130.55^T^) and clustered together. The large number of invariable fragments, together with the overall high similarity of >80% between the fingerprints, was in agreement with a monophyletic origin of the isolates. AFLP also clearly demarcated one set of isolates (VPCI 1562/10 and VPCI 1509/10), which represented clade 2 in *Cmd* tree and exhibited similar STR pattern. In addition, 2 *A*. *hortai* isolates forming clade 4 in *Cmd* tree, exhibited identical banding patterns and revealed less than 80% similarity with other *A*. *terreus* isolates. Also, the distinct group of isolates forming clade 5 in *Cmd* tree exhibited different banding pattern than the other groups suggesting a set of different genotypes. Additionally, a solitary isolate (VPCI 274/P/12) of clade 3 in *Cmd* tree fell in this AFLP group but exhibited differences in a few invariable bands. Similarly, type strain of *A*. *terreus* var. *floccosus* (syn. *A*. *floccosus*) was also represented by distinct banding profile suggesting AFLP could differentiate other species in *A*. *terreus* complex.

### Microsatellite typing

**Fig 3:**
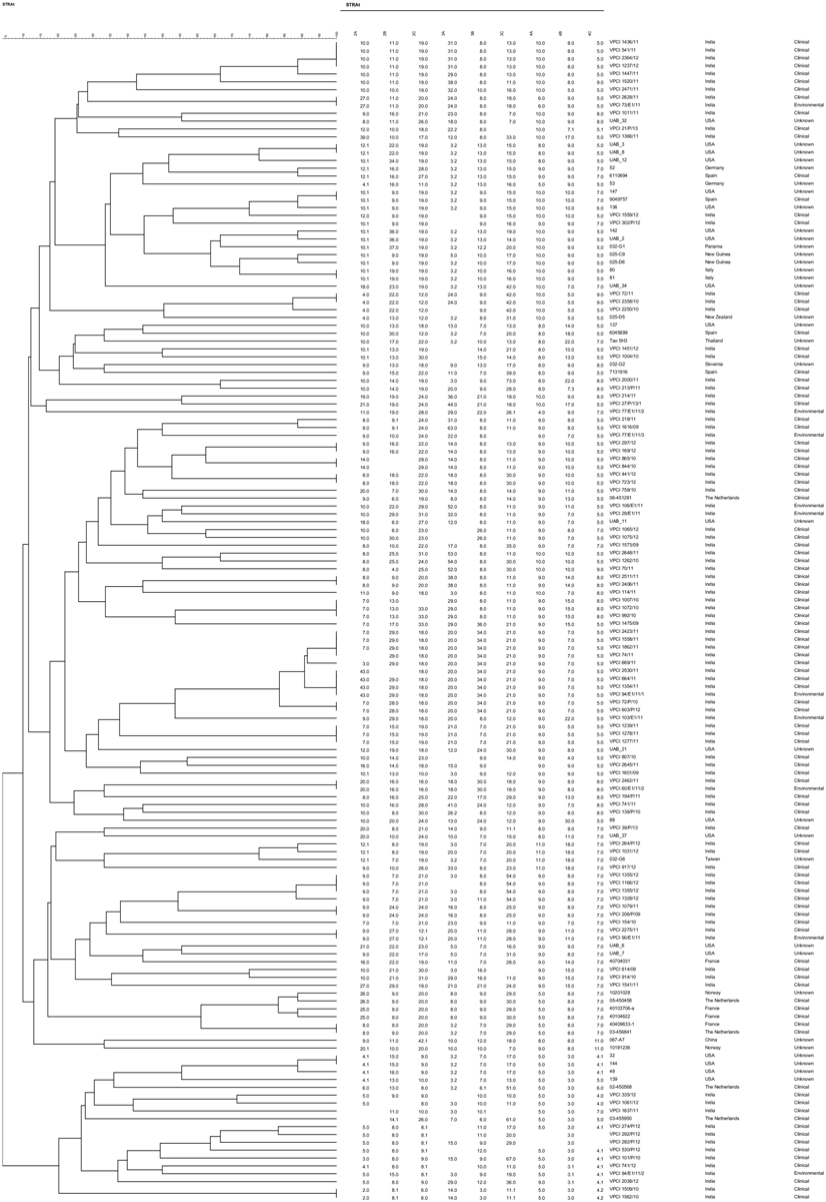
Analysis of genotypic relationship between *A*. *terreus* isolates from India (n = 101), Europe (20), USA (20), Panama (n = 1), New Zealand (n = 1), Papua New Guinea (n = 2), China (n = 1), Taiwan (n = 1) and Thailand (n = 1) using STR typing. The dendrogram is based on a categorical analysis of 9 microsatellite markers in combination with UPGMA clustering. The scale bar indicates the percentage identity.

**Fig 4:**
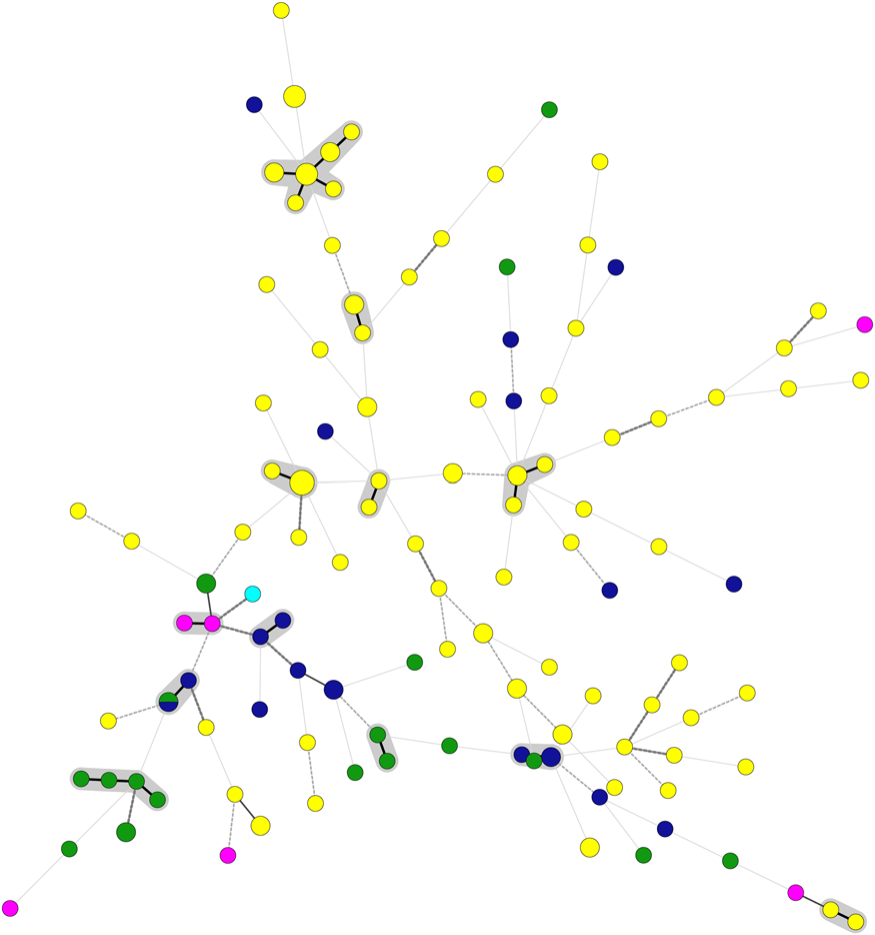
Minimum spanning tree showing wide genotypic diversity both in the clinical *A*. *terreus* isolates from India and those outside India. The figure shows the 115 different genotypes (circles), the number of strains belonging to the same genotype (sizes of the circles), and origin of isolates (circles in yellow indicate Indian isolates; green indicating European isolates including France (n = 4), Slovenia (n = 1), Germany (n = 2), Italy (n = 2), Norway (n = 2), Spain (n = 4), Netherlands (n = 5); pink indicate isolates from Australasia, including New Guinea (n = 2), New Zealand (n = 1), Taiwan (n = 1), China (n = 1), Thailand (n = 1); bright blue indicates isolate from Panama (Latin America; n = 1); dark blue indicates North American (n = 20) isolates). Gray-zone indicates microsatellite cluster representing minimal 2 isolates that differ maximum by 1 microsatellite marker out of 9. Thick and medium-thick branches indicate 1 or 2 microsatellite marker differences, respectively. Thick dashed line indicates 3 marker differences between two genotypes; 4 or more microsatellite markers differences between genotypes are indicated by medium thick and thin dashed lines, respectively.

The high genetic diversity of Indian *A*. *terreus* isolates among each other and with isolates from outside India was also observed in STR typing (Fig. 3). The STR typing of 101 Indian *A*. *terreus* isolates revealed 75 distinct genotypes distributed among environmental and clinical isolates. Similar marked heterogeneity was noticed in the global *A*. *terreus* population analyzed, which revealed unique 38 genotypes among 47 isolates. Among 10 distinct genotypes observed in 10 environmental Indian samples, 4 were also present in the clinical *A*. *terreus* population. Interestingly, the two isolates (VPCI 1509/10, VPCI 1562/10) that represented clade 2 in *Cmd* phylogenetic analysis and had 100% sequence similarity also showed an identical allelic profile at all the nine loci in STR typing. Noticeably, these two isolates revealed a new allelic profile at five of the nine loci studied which were not observed in the total population of *A*. *terreus* isolates analyzed. As mentioned above 21 isolates including 2 *A*. *hortai* (VPCI 906/12 and VPCI 317/P/11), could not be amplified after repeated attempts, and thus were excluded from the analysis. Notably, the STR profile of the 11 *A*. *terreus* isolates, which exhibited a distinct group in AFLP and *Cmd* phylogenetic tree (clade 5) revealed marked similarity at 5 loci in STR analysis. The analyses of the STR profile of all the *A*. *terreus* isolates including outside India revealed that loci 3A and 3C defined maximum heterogeneity with the large number of variable alleles. A marked genetic heterogeneity was observed in the minimum-spanning tree of Indian clinical *A*. *terreus* isolates (Fig. 4). Also, the tree clearly depicted no genotypic correlation of Indian *A*. *terreus* isolates with those outside India (Fig. 4).

### Antifungal susceptibility testing (AFST)

Overall, in the present study 8% (n = 11) of *A*. *terreus* isolates showed low AMB MICs ranging from 0.5–1 mg/L. All other isolates revealed AMB MICs ranging from 2->16 mg/L. Echinocandins, MFG (GM MEC, 0.015 mg/L), AFG (GM MEC, 0.016 mg/L) and CFG (GM MEC, 0.048 mg/L) exhibited highest activity against all the test isolates followed by POS (GM MIC, 0.07 mg/L) and ISA (GM MIC, 0.21 mg/L) (Table 1). In addition, ITC (GM MIC, 0.23 mg/L) and VRC (GM MIC 0.4 mg/L) were also active against the isolates. Also, 2 *A*. *hortai* isolates were susceptible to all the antifungals tested except AMB, which revealed MIC of 4–16 mg/L.

**Table 1:** *In- vitro* antifungal susceptibility profile of *Aspergillus terreus* and *A*. *hortai* isolates (n = 140) from India against medical triazoles, echinocandins and amphotericin B.

Species tested	MIC/MEC# parameters (mg/L)*	Drugs^#^							
		ITC	VRC	ISA	POS	AMB	CFG^$^	MFG^$^	AFG^$^
***A. terreus* (n = 138)**	**GM**	0.23	0.40	0.21	0.07	2.97	0.048	0.015	0.016
	**MIC_50_/MEC_50_^$^**	0.25	0.5	0.25	0.06	2	0.06	0.015	0.015
	**MIC_90_/MEC_90_^$^**	0.5	0.5	0.5	0.25	8	0.125	0.015	0.015
	**Range**	0.03–2	0.25–1	0.06–2	0.015–1	0.5–16	0.015–1	0.015–0.03	0.015–0.125
***A. hortai* (n = 2)**	**Range**	0.25–0.5	0.5	0.25–0.5	0.06–0.25	4–16	0.015–0.06	0.015	0.015

*MIC, minimum inhibitory concentration; MEC, minimum effective concentration was recorded for 3 echinocandins; GM, geometric mean; MIC_50_ andMIC_90_, MIC at which 50% and 90% of test isolates were inhibited respectively.

^$^MEC_50_ and MEC_90_, MEC at which 50% and 90% of test isolates revealed the growth of small, rounded, degenerated hyphae respectively.

^#^ITC, itraconazole; VRC, voriconazole; ISA, isavuconazole; POS, posaconazole; AMB, amphotericin B; CFG, caspofungin; MFG, micafungin; AFG, anidulafungin

### Clinical Summary

Out of 128 patients, *A*. *terreus* was a colonizer in 70 (54.6%) patients with chronic respiratory disorders such as chronic obstructive pulmonary diseases (COPD), interstitial lung disease, post-tubercular sequelae, and asthma involving both structurally damaged and intact lungs. In 58 (45.3%) patients, the fungus was implicated in the etiology of the disease. Among these, ABPA was diagnosed in 24 (41.3%) patients, IA in 13 (22.4%), aspergilloma in 10 (17.2%), CPA in 8 (13.7%) and AFRS in 3 (5.1%) patients. In the 24 cases with ABPA, 12 patients had both *A*. *fumigatus* and *A*. *terreus*-specific precipitins and both fungi were isolated in the sputum culture. However, specific IgE was significantly elevated for both the fungi in 9 of the 12 cases. Among IA patients, the two most common underlying conditions were hematological malignancies and COPD in 38.4% (n = 5) and 30.7% (n = 4) cases, respectively. The remaining patients included renal transplant recipients (n = 2, 15.3%) and those with interstitial lung diseases (n = 2, 15.3%). The COPD patients who developed IA had severe obstruction to airflow and required multiple admissions in the ward and intensive care units. These patients were on systemic and inhaled steroids for a long period ranging from 6–10 years. Moreover, majority of these COPD patients had associated comorbidities like diabetes mellitus (60%). The majority of IA cases were invasive pulmonary aspergillosis (85.7%). Both the IA cases, which had acute myeloid leukemia, developed breakthrough infections while receiving antifungal prophylaxis. The mortality in IA patients was high (85.7%) and was attributed to *A*. *terreus* infection in 75% cases. All the patients harboring an aspergilloma had post tubercular intrapulmonary cavities and *A*. *terreus* coexisted with *A*. *fumigatus* in two patients. The majority of the patients with ABPA (n = 16) were treated with systemic steroids and the remaining 8 were administered ITC for 6 weeks along with steroids. Seven of the 13 IA patients received VRC for 4–6 weeks whereas 3 patients were initially treated with AMB deoxycholate for two weeks followed by VRC for the next 3–4 weeks. In the remaining 3 cases, death occurred after 3 days of antifungal therapy due to the associated illnesses. In CPA patients, serum was positive for precipitating antibodies against *A*. *terreus* but serum galactomannan was negative. All the 8 cases with CPA received VRC, 4 of the patients showed symptomatic improvement whereas 2 were lost to follow up and 2 died.

## Discussion

The present study characterized a large number of *A*. *terreus* isolates using STR typing. Marked heterogeneity among Indian, North American and European isolates was noted. In the past, RAPD-PCR based methods have revealed high strain diversity among clinical and environmental isolates implicating that nosocomial acquisition of this pathogen is highly unlikely [9, 35, 36]. However, techniques based on complex banding pattern analysis, have poor reproducibility and do not allow exchange of data for global comparison. Furthermore, a solitary study based on multilocus phylogenetic approach by using three loci revealed a new cryptic species, *A*. *alabamensis* in the *A*. *terreus* complex but this technique has limited utility in strain discrimination [18]. Although STR typing, based on species-specific microsatellite loci, is highly species-specific and, is well established for other *Aspergillus* species such as *A*. *fumigatus* and *A*. *flavus*, it has so far not been utilized to detect genotypic diversity in *A*. *terreus* isolates from different geographical locations. This is the largest series employing STR typing for geographically diverse *A*. *terreus* isolates. In the present study, 19 *A*. *terreus* and 2 *A*. *hortai* isolates could not be amplified by the *A*. *terreus* loci revealing that other loci may have to be targeted for typing other species in the *A*. *terreus* complex. Furthermore in the present study a high concordance was observed in clustering the related isolates between AFLP typing and *Cmd* phylogenetic analysis. Also, AFLP technique proved to be both a typing as well as identification technique for *A*. *terreus* complex isolates. Notably, this technique has been reported to have a high discriminatory power and the robustness to serve as a powerful taxonomic tool for identification of isolates at the species level.

We report for the first time the isolation of *A*. *hortai*, from clinical cases of aspergillosis. This species comprised 1.4% of all *A*. *terreus* isolates identified by *Cmd* sequencing. *Aspergillus hortai* was described for the first time by Langeron in 1922 from the human ear and was considered a synonym for *A*. *terreus* by Raper and Fennel (1965) [37, 38]. However, Samson et al., [14] in 2011 clearly showed it to be distinct from *A*. *terreus* using a polyphasic approach and proposed it as a distinct species. Although this species reveals a strong morphological resemblance to *A*. *terreus*, it clearly exhibits a distinct extrolite profile. In the present study *A*. *hortai* isolates originated from broncho-alveolar lavage of cases of aspergilloma and probable IA. Previously the cryptic species *A*. *niveus* and *A*. *alabamensis* have been reported from a case of IA and as colonizers, respectively [18, 39]. The isolation of *A*. *hortai* in the present study from a case of fungal ball and IA extends the spectrum of other cryptic species in the *A*. *terreus* species complex. Although both *A*. *hortai* isolates exhibited 100% sequence similarity of the *Cmd* gene and identical banding pattern by AFLP analysis they originated from 2 different patients admitted in different hospitals of Delhi. Furthermore, the 2 *A*. *hortai* isolates exhibited genetic diversity from a solitary clinical Brazilian isolate and 2 environmental Galapagos Island *A*. *hortai* isolates.

Microsatellite typing is a useful, reproducible and discriminatory technique for strain typing of *Aspergillus* species [19, 26, 40, 41]. Indeed, 75 distinct genotypes were observed in the present collection of 101 Indian *A*. *terreus* isolates from clinical and environmental sources. Similarly, geographically diverse isolates from outside India analyzed by STR in the present study revealed no evidence of clonality among the population. All the nine markers used to type *A*. *terreus* species complex proved to be highly polymorphic displaying highest numbers of 34 and 32 alleles at loci 3A and 3C respectively, whereas a limited allelic variation (n = 8) was observed at locus 4A. The hypothesis of non endemicity of *A*. *terreus* isolates in a particular geographic location has been previously reported by Lass-Flörl et al. [8] and Blum et al. [9]. The authors in the former study analyzed 33 and 26 consecutive *A*. *terreus* isolates from patients with IA and hematological malignancies, respectively obtained from 2 geographically disparate institutions, namely, The University of Texas M. D. Anderson Cancer Center, Houston and the University Hospital of Innsbruck, Austria [8]. The analysis of RAPD profiles of all the *A*. *terreus* isolates resulted in 33 distinct profiles in the collection. No strain similarity between the two collections was observed, indicating great genomic diversity of *A*. *terreus* [8]. Similarly, Blum et al. [9] analyzed 49 *A*. *terreus* isolates from the environment and from aspergillosis patients at the University Hospital of Innsbruck. Genotypic analyses of *A*. *terreus* isolates with RAPD PCR revealed 46 distinct genotypic profiles among the environmental and clinical isolates, suggesting that the *A*. *terreus* population is genotypically diverse and lack any phylogeographic endemicity. However, in contrast a more recent study based on ISSR PCR for typing of *A*. *terreus* isolates revealed global sub-clustering of genotypes among *A*. *terreus* isolates and suggested a population structure linked to geographical origin in *A*. *terreus* [19]. Considering the inherent drawbacks of reproducibility of ISSR and RAPD, the latter method has been widely used for investigating the clonality of *A*. *terreus* isolates. The present study using a more robust STR technique for typing and strain discrimination of clinical and environmental isolates strengthens the evidence of lack of endemism among the *A*. *terreus* population. The genetic heterogeneity of all 10 environmental isolates investigated in the present study is similar to the RAPD analysis of 9 environmental isolates collected from the University Hospital of Cologne, revealing no clonal relationship among *A*. *terreus* isolates [35]. Overall, the high degree of genetic diversity obtained using both microsatellite and AFLP analyses in the present study is in excellent agreement with a sexual mode of reproduction in *A*. *terreus* [42]. Furthermore, no apparent correlation between genotypes and clinical presentation of the disease was observed in the present study. Probably, as previously reported in cases of aspergillosis due to *A*. *fumigatus*, host and other factors like environmental exposure, extensive use of antifungals for prophylaxis etc. play a more important role in the clinical presentation of disease than the genotypes of the isolates involved [43, 44].

Although, *A*. *terreus* is known for intrinsic resistance to AMB, 12–13% isolates with low AMB MICs have been observed worldwide in a few previous series, suggesting a possible genetic difference that may exist in this *Terrei* section complex [6, 7]. The present study revealed that 8% (11/140) of the *A*. *terreus* isolates exhibiting lower MICs of AMB (0.5–1 mg/L) were not limited to a particular genotypic pattern. Similarly, a small relationship between population structure and AMB susceptibility was reported among 145 clinical *A*. *terreus* isolates from Europe (n = 98) and the USA (n = 47) [19, 45]. Owing to the intrinsic resistance of *A*. *terreus* to AMB, data from *in vitro*, animal and clinical studies suggests that AMB is not an effective option for *A*. *terreus* infections [46, 47]. The treatment strategies such as the use of expanded-spectrum triazoles like VRC as a first line treatment and POS as prophylaxis and salvage therapy are recommended for IA [48, 49]. The present study observed higher potency of both VRC (GM MIC, 0.4 mg/L) and POS (GM MIC, 0.07 mg/L). Notably, only 3% of the global collection of clinical *A*. *terreus* isolates had MICs of VRC greater than the ECVs [50]. This is in concordance with the VRC MICs in the present study where none of the isolate had VRC MICs above ECVs. Furthermore, therapy with azoles in our patient population was effective especially in ABPA and CPA patients. In the former group, 8 patients treated with ITC showed no relapse after 6–12 months of follow up. Likewise, cases of fungal ball with hemoptysis in our study were successfully managed with VRC and have been asymptomatic for the last 9 months. The efficacy of VRC in prolonging the survival and reducing the fungal load in a murine model infected by *A*. *terreus* strains that showed MICs less than or equal to ECV has been recently reported [48]. However, emergence of azole resistance in *A*. *terreus* isolates in cystic fibrosis patient with elevated MICs for VRC and POS involving M217I alteration in *cyp51a* gene has been observed in a Danish patient [51].

Finally, considering that *A*. *terreus* causes fulminant infections, which are resistant to AMB therapy, the knowledge of genetic relatedness of *A*. *terreus* isolates, is of paramount importance to determine the source of infections and to study the population structure of this pathogen. In conclusion, high resolution typing method such as AFLP and microsatellite analysis in the present study yielded better understanding of the molecular epidemiology of *A*. *terreus* complex.

## Supporting Information

S1 Table (DOC)

S2 Table (DOC)
