# Supplementary material for: Molecular Epidemiology and In-Vitro Antifungal Susceptibility of Aspergillus terreus Species Complex Isolates in Delhi, India: Evidence of Genetic Diversity by Amplified Fragment Length Polymorphism and Microsatellite Typing
Source: PLoS One. 2015 Mar 17;10(3):e0118997. doi: 10.1371/journal.pone.0118997 (PMC4363790; doi:10.1371/journal.pone.0118997)
Supplement: S1 Table — (DOC) [file pone.0118997.s001.doc]

**Table S1. Details of Indian *A. terreus* isolates originating from clinical and environmental sources**

| **VPCI Accession nos.** | **Year of Isolation** | **GenBank Accession No.** | **Nature of clinical specimen** | **STR genotype** | **Underlying disorder** |
| --- | --- | --- | --- | --- | --- |
| 1475/09 | 2009 | KM386697 | FNAB | 7-17-33-29-36-21-9-15-5 | IA |
| 1555/09 | 2009 | KM458096 | Endotracheal aspirate | NA | ILD |
| 1573/09 | 2009 | KM386698 | BAL | 8-10-22-17-8-35-9-7-7 | ABPA |
| 1590/09 | 2009 | KM458097 | Endotracheal aspirate | NA | Aspergilloma |
| 1601/09 | 2009 | KM386699 | Endotracheal aspirate | 10.1-13-10-3-9-12-9-9-5 | CPA |
| 1616/09 | 2009 | KM386700 | Induced sputum | 8-9.1-24-63-8-11-9-8-5 | COPD |
| 206/P/09 | 2009 | KM386701 | BAL | 9-24-24-16-8-25-9-8-7 | ABPA |
| 614/09 | 2009 | KM386696 | Endotracheal aspirate | 10-21-30-3-16-X-9-15-7.0 | Aspergilloma |
| 1004/10 | 2010 | KM386709 | Endotracheal aspirate | 10.1-13-30-X-15-14-8-13-5 | ILD |
| 101/P/10 | 2010 | KM458100 | Induced sputum | 3-8-9-15-9-67-5-3-4.1 | COPD |
| 1262/10 | 2010 | KM386712 | Sputum | 8-25-24-54-8-30-10-10-5 | ILD |
| 138/P/10 | 2010 | KM386716 | Bronchial aspirate | 10-8-30-26.2-8-12-9-8-8 | CPA |
| 1509/10 | 2010 | KM458098 | Induced sputum | 2-8.1-6-14-3-11.1-5-3-4.2 | ILD |
| 154/10 | 2010 | KM386702 | BAL | 7-7-21-23-9-11-9-7-7 | IA |
| 1562/10 | 2010 | KM458099 | Induced sputum | 2-8.1-6-14-3-11.1-5-3-4.2 | Asthma |
| 2250/10 | 2010 | KM386713 | FNAB | 4-22-12-X-9-42-10-5-5 | IA |
| 2358/10 | 2010 | KM386714 | BAL | 4-22-12-24-9-42-10-5-9 | ABPA |
| 72/P/10 | 2010 | KM386715 | BAL | 7-28-18-20-34-21-9-7-5 | CPA |
| 759/10 | 2010 | KM386703 | Induced sputum | 20-7-30-14-8-14-9-11-5 | COPD |
| 807/10 | 2010 | KM386704 | Bronchial aspirate | 10-14-23-X-9-14-9-4-5 | Aspergilloma |
| 865/10 | 2010 | KM386706 | Sputum | 14-X-29-14-8-11-9-10-5 | ILD |
| 914/10 | 2010 | KM386707 | BAL | 10-21-31-29-16-11-9-15-7 | CPA |
| 992/10 | 2010 | KM386708 | FNAB | 7-13-33-29-8-11-9-15-8 | IA |
| 1007/10 | 2010 | KM386710 | Induced sputum | 7-13-33-29-8-11-9-15-8 | ILD |
| 1072/10 | 2010 | KM386711 | Endotracheal aspirate | 7-13-33-29-8-11-9-15-8 | Post tuberculosis cavitory lesions |
| 844/10 | 2010 | KM386705 | Sputum | 14-X-29-14-8-11-9-10-5 | ILD |
| 219/11 | 2011 | KM386724 | BAL | 14-X-29-14-8-11-9-10-5 | Aspergilloma |
| 2471/11 | 2011 | KM386750 | Induced sputum | 10-10-19-32-10-16-10-5-5 | Asthma |
| 2645/11 | 2011 | KM386754 | Sputum | 16-14-18-15-9-X-9-9-5 | ILD |
| 2648/11 | 2011 | KM386755 | Induced sputum | 8-25-31-53-8-11-10-10- 5 | Asthma |
| 317/P/11 | 2011 | KM386817 | BAL | NA | CPA |
| 669/11 | 2011 | KM386728 | Endotracheal Aspirate | 3-29-18-20-34-21-9-7-5 | Aspergilloma |
| 72/11 | 2011 | KM386718 | Sputum | 4-22-12-24-9-42-10-5-9 | Asthma |
| 74/11 | 2011 | KM386719 | FNAB | X-29-18-20-34-21-9-7-5 | IA |
| 741/11 | 2011 | KM386729 | BAL | 10-16-28-41-24-12-9-7-8 | CPA |
| 1345/11 | 2011 | KM386735 | Sputum | NA | ILD |
| 2423/11 | 2011 | KM386748 | Induced sputum | 7-29-18-20-34-21-9-7-5 | COPD |
| 2462/11 | 2011 | KM386749 | BAL | 20-16-16-18-30-18-9-8-8 | CPA |
| 2511/11 | 2011 | KM386751 | Sputum | 8-9-20-38-8-11-9-14-8 | COPD |
| 2530/11 | 2011 | KM386752 | FNAB | 43-X-18-20-34-21-9-7-5 | IA |
| 2628/11 | 2011 | KM386753 | BAL | 27-11-20-24-8-18-6-9-5 | ABPA |
| 440/11 | 2011 | KM386725 | Induced sputum | ND | COPD |
| 541/11 | 2011 | KM386726 | Endotracheal aspirate | 10-11-19-31-8-13-10-8-5 | ILD |
| 664/11 | 2011 | KM386727 | FNAB | 43-X-18-20-34-21-9-7-5 | IA |
| 70/11 | 2011 | KM386717 | BAL | 8-4-25-52-8-30-10-10-9 | ABPA |
| 114/11 | 2011 | KM386720 | Induced sputum | 11-9-18-3-8-11-10-7-8 | ILD |
| 1277/11 | 2011 | KM386733 | Sputum | 7-15-19-21-7-21-9-5-5 | ILD |
| 198/11 | 2011 | KM386722 | Sputum | NA | Asthma |
| 154/11 | 2011 | KM386721 | Induced sputum | NA | Asthma |
| 1541/11 | 2011 | KM386741 | Endotracheal aspirate | 27-29-19-21-21-24-9-15-7 | Post tubercular cavitory lesions |
| 1862/11 | 2011 | KM386744 | Sputum | 7-29-18-20-34-21-9-7-5 | Asthma |
| 1011/11 | 2011 | KM386730 | BAL | 9-16-21-23-8-7-10-9-8 | ABPA |
| 1079/11 | 2011 | KM386731 | Sputum | 9-24-24-16-8-25-9-8-7 | Post tubercular cavitory lesions |
| 1239/11 | 2011 | KM386732 | FNAB | 7-15-19-21-7-21-9-5-5 | IA |
| 1278/11 | 2011 | KM386734 | Bronchial aspirate | 7-15-19-21-7-21-9-5-5 | CPA |
| 1354/11 | 2011 | KM386736 | BAL | 43-X-18-20-34-21-9-7-5 | ABPA |
| 1366/11 | 2011 | KM386737 | Nasal wash | 39-10-17-12-8-33-10-17-5 | AFRS |
| 1436/11 | 2011 | KM386738 | Sputum | 10-11-19-31-8-13-10-8-5 | Post tubercular |
| 1447/11 | 2011 | KM386739 | FNAB | 10-11-19-29-8-13-10-8-5 | IA |
| 1520/11 | 2011 | KM386740 | Induced sputum | 10-11-19-31-8-13-10-8-5 | Post tubercular |
| 1558/11 | 2011 | KM386742 | Nasal wash | 7-29-18-20-34-21-9-7-5 | AFRS |
| 1637/11 | 2011 | KM386743 | BAL | X-11-10-3-10.1-X-5-3-7 | ABPA |
| 194/P/11 | 2011 | KM386756 | Endotracheal aspirate | 8-16-25-22-17-29-9-13-8 | Asthma |
| 2000/11 | 2011 | KM386745 | Induced sputum | 10-14-19-3-9-73-8-22-8 | COPD |
| 213/P/11 | 2011 | KM386757 | BAL | 10-14-19-20-9-28-8-7.3-8 | ABPA |
| 214/11 | 2011 | KM386723 | Sputum | 19-19-24-36-21-18-10-9-8 | ILD |
| 2275/11 | 2011 | KM386746 | FNAB | 9-27-12.1-20-11-28-9-11-7 | IA |
| 2406/11 | 2011 | KM386747 | Induced sputum | 8-9-20-38-8-11-9-14-8 | ILD |
| 723/12 | 2012 | KM386773 | BAL | 8-18-22-18-8-30-9-10-5 | ABPA |
| 1061/12 | 2012 | KM458104 | Bronchial aspirate | 5-X-8-3-10-11-5-3-4 | Aspergilloma |
| 1065/12 | 2012 | KM386777 | BAL | 10-6-23-X-26-11-9-8-7 | ABPA |
| 1075/12 | 2012 | KM386778 | Endotracheal aspirate | 10-30-26-X-26-11-9-7-5 | Asthma |
| 1130/12 | 2012 | KM386779 | Sputum | NA | Post tubercular |
| 1166/12 | 2012 | KM458105 | Endotracheal aspirate | 9-7-21-X-8-54-9- 8-7 | ILD |
| 1181/12 | 2012 | KM386780 | Induced sputum | ND | ILD |
| 1237/12 | 2012 | KM386781 | Sputum | 10-11-19-31-8-13-10-8-5 | Asthma |
| 1247/12 | 2012 | KM386782 | Induced sputum | ND | Post tubercular |
| 1281/12 | 2012 | KM458106 | Induced sputum | ND | Asthma |
| 1328/12 | 2012 | KM458107 | Sputum | 9-7-21-3-11-54-9-8-7 | Asthma |
| 1355/12 | 2012 | KM386783 | BAL | 9-7-21-3-8-54-9-8-7 | ABPA |
| 1451/12 | 2012 | KM386784 | Bronchial aspirate | 10.1-13-19-X-14-21-8-10-5 | Aspergilloma |
| 1559/12 | 2012 | KM386785 | Sputum | 12-9-19-X-9-15-10-10-5 | Post tubercular |
| 1634/12 | 2012 | KM386786 | Endotracheal aspirate | ND | ILD |
| 1674/12 | 2012 | KM386787 | Sputum | ND | Asthma |
| 169/12 | 2012 | KM386769 | FNAB | 9-16-22-14-8-13-9-10-5 | IA |
| 2038/12 | 2012 | KM458108 | BAL | 5-8-9-29-12-36-9-3.1-4.1 | ABPA |
| 221/12 | 2012 | KM386770 | Endotracheal aspirate | ND | ILD |
| 264/P/12 | 2012 | KM386790 | Endotracheal aspirate | 12-8-19-3-7-20-11-18-7 | Asthma |
| 274/P/12 | 2012 | KM458109 | BAL | 5-8-8.1-X-11-17-5-3-4.1 | ABPA |
| 282/P/12 | 2012 | KM458110 | FNAB | 5-8-8.1-15-9-29-X-3-X | IA |
| 292/P/12 | 2012 | KM458111 | Induced sputum | 5-8-8.1-X-11-20-X-3-X | Post tubercular lung damage |
| 297/12 | 2012 | KM386771 | Induced sputum | 9-16-22-14-8-13-9-10-5 | Asthma |
| 302/P/12 | 2012 | KM386791 | BAL | 10.1-9-19-X-9-16-9-9-7 | ABPA |
| 335/12 | 2012 | KM458102 | Induced sputum | 5-9-9-X-10-10-5-30-4 | Post tubercular lung damage |
| 441/12 | 2012 | KM386772 | BAL | 8-18-22-18-8-30-9-10-5 | ABPA |
| 530/P/12 | 2012 | KM458112 | Nasal wash | 5-8-9.1-X-12-5-3-4.1 | Aspergilloma |
| 603/P/12 | 2012 | KM386792 | FNAB | 7-28-18-20-34-21-9-7-5 | ABPA |
| 741/12 | 2012 | KM458103 | Sputum | 4.1-8-8.1-X-10-11-5-3.1-4.1 | COPD |
| 906/12 | 2012 | KM386818 | BAL | NA | ABPA |
| 917/12 | 2012 | KM386774 | Endotracheal aspirate | 9-10-26-33-8-23-11-18-7 | COPD |
| 1031/12 | 2012 | KM386776 | BAL | 12.1- 8-19- 20- 7-20- 11-18-7 | ABPA |
| 2108/12 | 2012 | KM386788 | Induced sputum | NA | Post tubercular cavitory lesions |
| 2364/12 | 2012 | KM386789 | BAL | 10-11-19-31-8-13-10-8-5 | Aspergilloma |
| 1011/12 | 2012 | KM386775 | nasal mucosa biopsy | NA | AFRS |
| 49/13 | 2013 | KM386793 | BAL | ND | COPD |
| 1012/P/13 | 2013 | KM386812 | Induced sputum | NA | Post tubercular cavitory lesions |
| 104/13-B | 2013 | KM386794 | BAL | ND | COPD |
| 107/P/13 | 2013 | KM386806 | Endotracheal aspirate | ND | Asthma |
| 1086/13 | 2013 | KM386798 | FNAB | 7-28-18-20-34-21-9-7-5 | IA |
| 138/13 | 2013 | KM386795 | Induced sputum | ND | ILD |
| 1884/13 | 2013 | KM386800 | Sputum | ND | Post tubercular cavitory lesions |
| 21/P/13 | 2013 | KM386802 | BAL | 12-10-18-22.2-8-X-10-7.1-5.1 | ABPA |
| 2292/13 | 2013 | KM386801 | Endotracheal aspirate | ND | COPD |
| 27/P/13/1 | 2013 | KM386803 | Endotracheal aspirate | 21-19-24-44-21-18-10-17-5 | Asthma |
| 308/P/14 | 2014 | KM386813 | BAL | ND | ILD |
| 374/P/13 | 2013 | KM386807 | Induced sputum | NA | Post tubercular lung damage |
| 39/P/13 | 2013 | KM386804 | Induced sputum | 20-8-21-14-9-11.1-8-9-7 | ILD |
| 423/P/13 | 2013 | KM386808 | Sputum | NA | COPD |
| 550/P/13 | 2013 | KM386809 | BAL | NA | COPD |
| 672/13 | 2013 | KM386796 | Sputum | NA | Post tubercular cavitory lesions |
| 693/13 | 2013 | KM386797 | Induced sputum | NA | COPD |
| 820/P/13 | 2013 | KM386810 | Bronchial aspirate | NA | Aspergilloma |
| 947/P/13 | 2013 | KM386811 | Sputum | ND | Post tubercular |
| 98/P/13 | 2013 | KM386805 | FNAB | NA | ABPA |
| 1286/13 | 2013 | KM386799 | Induced sputum | ND | COPD |
| F 398 I | 2013 | KM386814 | BAL | NA | ABPA |
| F 404 III | 2013 | KM386815 | BAL | NA | ABPA |
| F 417 IV | 2013 | KM386816 | BAL | NA | ABPA |
| **Environmental** | | | | | |
| 60/EI/11/1_E | 2011 | KM386759 | Soil, Paddy field (Bodia, Haryana) | ND | - |
| 82/EI/11/1_E | 2011 | KM386764 | Soil, DU Central park | ND | - |
| 77/EI/11/3_E | 2011 | KM386763 | Soil, Red chilli, Delhi | 9-10-24-22-8-X-9-7-5 | - |
| 90/EI/11_E | 2011 | KM386765 | Soil, Rose garden, Delhi | 9-27-12.1-20-11-28-9-11-7 | - |
| 73/EI/11_E | 2011 | KM386761 | Soil, DU Central park | 27-11-20-24-8-18-6-9-5 | - |
| 106/EI/11_E | 2011 | KM386768 | Soil, Wheat, Haryana | 10-22-29-52-8-11-9-11-5 | - |
| 103/EI/11_E | 2011 | KM386767 | Soil, Fenugreek, Haryana | 9-29-18-20-8-12-9-22-5 | - |
| 77/EI/11/2_E | 2011 | KM386762 | Soil, Red chilli, Delhi | 11-19-28-29-22-26.1-4-9-7 | - |
| 94/EI/11/1_E | 2011 | KM386766 | Soil, Rose garden, Delhi | 43-X-18-20-34-21-9-7-5 | - |
| 94/EI/11/2_E | 2011 | KM458101 | Soil, Rose garden, Delhi | 5-15-8.1-3-9-19-5-3.1-4.1 | - |
| 29/EI/11_E | 2011 | KM386758 | Soil, Paddy field, Haryana | 10-29-31-32-8-11-9-7-5 | - |
| 60/EI/11/2_E | 2011 | KM386760 | Soil, Paddy field, Haryana | 20-16-16-18-30-18-9-8-8 | - |

Abbreviations used: FNAB, Fine needle aspiration biopsy; BAL, Broncho alveolar lavage; IA, Invasive aspergillosis; ILD, Interstitial lung disease; COPD, Chronic obstructive pulmonary disease; CPA, Chronic pulmonary aspergillosis; NA, Not amplified; ND, Not done.
