# Supplementary material for: Molecular Epidemiology and In-Vitro Antifungal Susceptibility of Aspergillus terreus Species Complex Isolates in Delhi, India: Evidence of Genetic Diversity by Amplified Fragment Length Polymorphism and Microsatellite Typing
Source: PLoS One. 2015 Mar 17;10(3):e0118997. doi: 10.1371/journal.pone.0118997 (PMC4363790; doi:10.1371/journal.pone.0118997)
Supplement: S2 Table — (DOC) [file pone.0118997.s002.doc]

**Table S2. Details of global *A. terreus* isolates used in the present study for STR analysis**

| **Accession nos.** | **Source** | **STR genotype** | **Geographic Origin of the isolates** |
| --- | --- | --- | --- |
| UAB32 | BAL | 8-11-26-18-8-7-10-9-8 | USA |
| UAB3 | Bronchial wash | 12.1-22-19-3.2-13-15-8-9-5 | USA |
| UAB 8 | Thyroid | 12.1-22-19-3.2-13-15-8-9-5 | USA |
| UAB12 | Sputum | 10.1-34-19-3.2-13-15-8-9-5 | USA |
| 52 | Unknown | 12.1-16-28-3.2-13-15-9-9-7 | Germany |
| 6110694 | Clinical | 12.1-16-27-3.2-13-15-9-9-7 | Spain |
| 53 | Unknown | 4.1-16-11-3.2-13-16-5-9-5 | Germany |
| 147 | Unknown | 10.1-9-19-3.2-9-15-10-10-7 | USA |
| 9049757 | Clinical | 10.1-9-19-3.2-9-15-10-10-7 | Spain |
| 136 | Unknown | 10.1-9-19-3.2-9-15-10-10-6 | USA |
| 142 | Unknown | 10.1-36-19-3.2-13-19-10-9-5 | USA |
| UAB2 | Sputum | 10.1-36-19-3.2-13-14-10-9-5 | USA |
| 032-G1 | Unknown | 10.1-37-19-3.2-12.2-20-10-9-5 | Panama |
| 025-C9 | Unknown | 10.1-9-19-5-10-17-10-9-5 | Papua New Guinea |
| 025-D6 | Unknown | 10.1-9-19-3.2-10-17-10-9-5 | Papua New Guinea |
| 80 | Unknown | 10.1-19-19-3.2-10-16-10-9-5 | Italy |
| 81 | Unknown | 10.1-19-19-3.2-10-16-10-9-5 | Italy |
| UAB34 | Sputum | 18-23-19-3.2-13-42-10-7-7 | USA |
| 025-D5 | Unknown | 4-13-12-3.2-8-31-10-5-5 | New Zealand |
| 137 | Unknown | 10-13-18-13-7-13-8-14-5 | USA |
| 6045699 | Clinical | 10-30-12-3.2-7-20-8-18-5 | Spain |
| Tao 5H3 | Unknown | 10-17-22-3.2-10-13-8-22-7 | Thailand |
| 032-G2 | Unknown | 9-13-18-9-13-17-8-9-8 | Slovenia |
| 7131916 | Clinical | 9-15-22-11-7-39-8-9-5 | Spain |
| 06-451281 | Clinical | 9-6-19-8-8-14-9-13-5 | The Netherlands |
| UAB11 | Sputum | 18-6-27-12-8-11-9-7-5 | USA |
| UAB21 | Sputum | 12-19-18-12-24-30-9-8-5 | USA |
| 88 | Unknown | 10-20-24-13-24-12-9-30-5 | USA |
| UAB37 | Tracheal aspirate | 20-10-24-10-7-15-8-11-7 | USA |
| 032-G6 | Unknown | 12.1-7-19-3.2-7-20-11-18-7 | Taiwan |
| UAB6 | Sputum | 21-22-23-5-7-16-9-9-7 | USA |
| UAB7 | Bronchial wash | 9-22-17-5-7-31-9-8-7 | USA |
| 40704031 | Clinical | 16-22-19-11-7-28-9-14-7 | France |
| 10201028 | Unknown | 26-9-20-8-9-29-5-8-7 | Norway |
| 05-450458 | Clinical | 26-9-20-8-9-30-5-8-7 | The Netherlands |
| 40103706-a | Clinical | 25-9-20-8-9-29-5-8-7 | France |
| 40104622 | Clinical | 25-9-20-8-9-30-5-8-7 | France |
| 40409633-1 | Clinical | 8-9-20-3.2-7-29-5-8-7 | France |
| 03-456841 | Clinical | 8-9-20-3.2-7-29-5-8-7 | The Netherlands |
| 067-A7 | Unknown | 9-11-42.1-10-12-18-8-8-11 | China |
| 10181236 | Unknown | 20.1-10-20-10-10-7-9-8-11 | Norway |
| 32 | Unknown | 4.1-15-9-3.2-7-17-5-3-4.1 | USA |
| 144 | Unknown | 4.1-15-9-3.2-7-17-5-3-4.1 | USA |
| 48 | Unknown | 4.1-16-9-3.2-7-17-5-3-4.1 | USA |
| 139 | Unknown | 4.1-13-10-3.2-7-13-5-3-5 | USA |
| 02-450568 | Clinical | 6-13-8-3.2-6.1-51-5-3-6 | The Netherlands |
| 03-455950 | Clinical | X-14.1-26-7-6-61-5-3-5 | The Netherlands |
